# Dog ownership and the risk of cardiovascular disease and death – a nationwide cohort study

**DOI:** 10.1038/s41598-017-16118-6

**Published:** 2017-11-17

**Authors:** Mwenya Mubanga, Liisa Byberg, Christoph Nowak, Agneta Egenvall, Patrik K. Magnusson, Erik Ingelsson, Tove Fall

**Affiliations:** 1 Department of Medical Sciences, Molecular Epidemiology and Science for Life Laboratory, Uppsala University, Uppsala, Sweden; 2 Department of Surgical Sciences, Orthopedics, Uppsala University, Uppsala, Sweden; 3 Department of Neurobiology, Care Sciences and Society, Karolinska Institutet, Huddinge, Sweden; 4 Department of Clinical Sciences, Division of Ruminant Medicine and Veterinary Epidemiology, Swedish University of Agricultural Sciences, Uppsala, Sweden; 5 Department of Medical Epidemiology and Biostatistics, Karolinska Institutet, Stockholm, Sweden; 6 Department of Medicine, Division of Cardiovascular Medicine, Stanford University School of Medicine, Stanford, CA USA

## Abstract

Dogs may be beneficial in reducing cardiovascular risk in their owners by providing social support and motivation for physical activity. We aimed to investigate the association of dog ownership with incident cardiovascular disease (CVD) and death in a register-based prospective nation-wide cohort (n = 3,432,153) with up to 12 years of follow-up. Self-reported health and lifestyle habits were available for 34,202 participants in the Swedish Twin Register. Time-to-event analyses with time-updated covariates were used to calculate hazard ratios (HR) with 95% confidence intervals (CI). In single- and multiple-person households, dog ownership (13.1%) was associated with lower risk of death, HR 0.67 (95% CI, 0.65–0.69) and 0.89 (0.87–0.91), respectively; and CVD death, HR 0.64 (0.59–0.70), and 0.85 (0.81–0.90), respectively. In single-person households, dog ownership was inversely associated with cardiovascular outcomes (HR composite CVD 0.92, 95% CI, 0.89–0.94). Ownership of hunting breed dogs was associated with lowest risk of CVD. Further analysis in the Twin Register could not replicate the reduced risk of CVD or death but also gave no indication of confounding by disability, comorbidities or lifestyle factors. In conclusion, dog ownership appears to be associated with lower risk of CVD in single-person households and lower mortality in the general population.

## Introduction

Cardiovascular disease (CVD) is the leading cause of death worldwide, accounting for 45% of all deaths (>4 million) in Europe in 2016^1,2^. Dogs may be beneficial in reducing cardiovascular risk by providing a non-human form of social support and increasing physical activity. Dog ownership has been reported to be associated with alleviation of social isolation and improved perception of wellbeing, particularly in single persons and the elderly^3–5^. A meta-analysis of eleven observational studies found that dog owners walked more and were more physically active than non-owners^6^. Two studies assessing changes in physical activity after acquisition of a dog or other pet found increased self-reported recreational walking^7,8^. A recent study showed that dog ownership also supports the maintenance of physical activity during poor weather^9^.

Previous findings on dog ownership and the risk of CVD are conflicting. In individuals without CVD, dog ownership has been reported as inversely associated with the prevalence of cardiovascular risk factors, such as dyslipidemia, hypertension and diabetes^10,11^, but other studies found absent or inconclusive associations^12,13^. In patients with coronary artery disease, dog ownership is reportedly associated with improved survival^14–16^.

Although the American Heart Association issued a *Scientific Statement* in 2013 concluding that “[…] dog ownership is probably associated with decreased CVD risk […]” and that this effect “[…] may be causal […]”^17^, earlier studies have had several limitations, including lack of power, low responder rates and incomplete adjustment for potential confounders.

We aimed to clarify the association of dog ownership with CVD and mortality by studying over 3.4 million Swedish adults followed through nationwide register linkage over a 12-year period.

## Methods

### Study population – National Cohort

All Swedish residents aged 40 to 80 years on January 1, 2001 (n = 3,987,937) were eligible for this study. The age range was chosen to exclude younger individuals at low risk of CVD and the elderly at low odds of owning a dog. Participants were identified using the Register of the Total Population, which contains information on birth, migration, changes of citizenship, civil status and death on all Swedish citizens and residents^18^. By using the unique 10-digit personal identity number, records were linked to national registers for socioeconomic variables and health outcomes (see Supplementary Material).

### Study population - Swedish Twin Register

To verify findings from the national cohort in a subgroup with more available information on additional potential confounders, we repeated our analysis in the “Screening Across the Lifespan Twin study” (SALT) sub-study of the Swedish Twin Register (STR). The STR, initiated in 1958, is a longitudinal study of the vast majority of twins born in Sweden after 1886^19^ and SALT is a telephone-based interview conducted between March 1998 and March 2002 that collected information on twins born in 1958 or earlier. Of the 44,821 (74%) responders, 41,039 were aged 42–80 years in 2001 and hence eligible for this study^20^.

### Exclusions

Individuals (n = 163,156) were excluded from the National Cohort if they had not resided continuously in Sweden since 1987 to ensure sufficient information on dog ownership registration and complete medical history. Those with re-used or unconfirmed personal identity numbers (n = 5,057) were also excluded.

All Swedish residents are covered by the public health care system, and all hospital visits are registered in the National Patient Register^21^. Individuals with inpatient visits (n = 387,571 in national cohort and n = 3,163 in twin cohort) between January 1, 1987 to December 31, 2000 for CVD (ICD-9 codes 390–459 or ICD-10 I00-I99; main or secondary diagnosis) and/or had a coronary artery bypass grafting or percutaneous coronary artery intervention (Nordic surgical procedure codes FNA, FNC and FNG) were excluded from the study The final data set included 3,432,153 participants in the national cohort and 34,202 in the twin cohort (Supplementary Figure 1).

### Exposure

Since January 1, 2001, it has been a statutory requirement that every dog in Sweden has a unique identifier (ear tattoo or subcutaneous chip) registered at the Swedish Board of Agriculture. In addition, the Swedish Kennel Club has registered all dogs with a certified pedigree with complete information on owner’s personal identity number since 2001^22^. In 2012, an estimated 83% (95% confidence interval, CI, 78–87%) dogs were registered in at least one of these two registers^23^.

Dog ownership was defined as periods registered or having a partner registered as a dog owner in either of the two dog registers (Supplementary Figure 2). The linkage to each respective partner (defined as a married couple or a cohabiting couple with common children) was possible through annual extracts from the Register of the Total Population. If information on dog death was missing, we assumed a maximum lifespan of ten years. If dates were discrepant between the two registers, we randomly selected one of the two. We also used the Swedish Kennel Club’s definition of breed groups to categorize the 339 breeds into ten groups (Supplementary Table 1). All non-purebred dogs and those of unknown breeds were grouped as mixed breed.

In the twin data, we did not have access to information on partners’ dog ownership and only each person’s own dog registrations were used.

### Outcomes

We obtained death data from the Cause of Death Register^24^ and incident disease data from the National Patient Register from January 1, 2001 to December 31, 2012 in the national cohort, and up to December 31, 2014 in the twin cohort. In addition to all-cause mortality, the main diagnosis in inpatient and outpatient care and underlying cause of death were used to define four incident disease outcomes: (1) acute myocardial infarction (ICD-10 I21); (2) heart failure (ICD-10 I50); (3) ischemic stroke (ICD-10 I63) and (4) hemorrhagic stroke (ICD-10 I60-I62). Any occurrence of these diagnoses was additionally considered as a composite CVD outcome. CVD mortality was defined as death with the underlying cause being any of the CVD outcomes defined above. In the twin register only the composite CVD outcome and all-cause mortality were assessed.

### Covariates

Theoretical structured ordering of factors was undertaken by use of directed acyclic graphs to identify potential confounders (Supplementary Figure 3). Socioeconomic variables were accessed from annual excerpts of the Register of the Total Population and the Longitudinal Integration Database for Health Insurance and Labor Market Studies. Variables assessed only at baseline included sex, year of birth, country of birth (Sweden, other Nordic countries and non-Nordic countries) and educational attainment (compulsory, ≤9 years; secondary, 10–11 years; or tertiary education, ≥12 years; not available for those aged >75 years). Time-updated covariates included marital status (single, married/cohabiting, divorced or widowed: definition in Supplementary Methods), presence of children in the home (yes/no), area of residence (Norrland, Götaland and Svealand), population density in municipality of residence (continuous variable), and annual household income (birth year-standardized quintiles). We further accounted for a north-south gradient by including the latitude of the municipality of residence. We further added a time-updated variable for two variants of the socioeconomic index, the international socio-economic index (ISEI)^25^ and the European Socioeconomic index (ESeC)^26^ in those with sufficient information (Supplementary Methods).

To avoid reverse effects of outcomes on covariates, we used covariate data from the preceding year to time-update information on January 1 in every year. A stratification variable for household was created with individuals assigned to ‘single-person household’ if the participant lived alone, or to ‘multiple-person household’ if they were married, living with a partner and/or a child. Non-married partners are only traceable in the registers if the couple has children together. A second stratification variable was created for age group in decades.

In the twin register, no register data on socioeconomic and demographic covariates were available. Instead, the following self-reported variables at baseline were used (Supplementary Table 2): body mass index (BMI), physical activity (less than average, average, more than average), employment status (employed, retired, long-term sick leave/early retirement, unemployed), type of accommodation (independent housing, assisted living, other), smoking status (never, former, current), any physical impairment, requirement of assistance for routine daily activities and socioeconomic index (unskilled employees, lower skilled non-manual employees, self-employed excluding independent occupations, intermediate non-manual workers and higher non-manual workers). Data from the National Patient Register from January 1 1996 to December 31 2000 were used to calculate Charlson’s Comorbidity Index^27,28^ (Supplementary Table 3).

### Statistical Analysis

Cox proportional hazards regression with attained age as time-scale was used to calculate hazard ratios (HR) and 95% confidence intervals (CI) for each of the seven outcomes separately. The proportional hazards assumption was verified using log-log plots and Schoenfield residuals plots. Participants were censored at emigration (available in national cohort only), death or at the end of the study; whichever came first. For each outcome, we estimated one sex-adjusted model and one multivariable adjusted model.

#### National cohort

The multivariable-adjusted model included sex, country of birth, marital status, presence of children in the home, area of residence, population density, age-adjusted income and latitude. All analyses were implemented in the full study sample and stratified on household-type (single/multiple person), age group in decades and sex. In sensitivity analysis, we added adjustment for education in those <75 years at baseline and adjustment for ISEI and ESeC in those with sufficient information. Additionally, we tested for associations between different dog breed groups and outcomes.

#### Swedish Twin Register

The first multivariable model was similar to the national cohort model with socioeconomic index replacing age-adjusted income. All analyses were implemented in the full study sample and stratified on household type. In additional models, further adjustments for body mass index, smoking, Charlson Comorbidity Index, employment status and level of exercise was done. We repeated analyses after excluding those residing in assisted living accommodation, those with any physical impairments and those requiring assistance for routine daily activities. Standard errors were adjusted with the robust sandwich estimator for dependent observations.

Analyses were conducted in Stata/ MP 14.1 (StataCorp LP, TX, US). The study was approved by the Regional Ethical Review Board in Stockholm, Sweden (2012/1114-31/2 with amendments 2013-1687-32 and 2016/1392-31/1) and allowed the researchers to waive the requirement for obtaining informed consent in the national register part of the study. Participants in the SALT (Twin register) had given informed consent. The study was performed in accordance with relevant guidelines and regulations.

### Data availability statement

The register data that support the findings of this study were made available by record-linkage with data from Statistics Sweden, the National Board of Health and Welfare, the Swedish Kennel Club, Swedish Board of Agriculture and the Swedish Twin Register. Restrictions apply to the availability of these data, which were used under license and ethical approval for the current study, and so are not publicly available. Data are however available from the authors upon reasonable request and with permission of the Regional Ethical Review Board in Stockholm, Sweden.

## Results

### National cohort

The final sample included 3,432,153 individuals (48% men) with a mean age of 57 years. We identified 13.1% as dog owners at any time during the 12-year study period. Baseline characteristics on January 1 2001 are shown in Table 1. Dog owners were younger than non-owners at baseline (mean age, 52 vs. 58 years) and more likely to reside in areas of lower population density (median 57 inhabitants per km^2^ vs. 74 inhabitants per km^2^).

**Table 1:** Baseline characteristics of Swedish adults aged 40–80 years without cardiovascular disease on 1 January 2001 from the Register of the Total Population (national cohort, n = 3,432,153) and the Swedish Twin Register (twin cohort, n = 34,202).

	National cohort			Twin cohort		
	Dog owners^4^ n = 448,298 (13.1%)	Non-dog owners n = 2,983,855 (86.9%)	All n = 3,432,153 (100%)	Dog owners n = 2,909 (8.5%)	Non-dog owners n = 31,293 (91.5%)	All n = 34,202 (100%)
Age - mean ± SD	51.7 ± 8.2	57.9 ± 11.1	57.1 ± 11.0	53.3 ± 7.7	57.8 ± 9.8	57.4 ± 9.7
Male	219,105 (48.9%)	1,419,396 (47.6%)	1,638,501 (47.7%)	1,149 (39.5%)	14,179 (45.3%)	15,328 (44.8%)
**Marital status**						
Married or cohabiting	355,882 (79.4%)	1,863,429 (62.5%)	2,219,311 (64.7%)	2,301 (79.1%)	23,608 (75.4%)	25,909 (75.8%)
Never married	33,470 (7.5%)	417,700 (14.0%)	451,170 (13.1%)	250 (8.6%)	3,016 (9.6%)	3,266 (9.5%)
Divorced	49,766 (11.1%)	463,259 (15.5%)	513,025 (14.9%)	274 (9.4%)	3,006 (9.6%)	3,280 (9.6%)
Widowed	9,180 (2.0%)	239,467 (8.0%)	248,647 (7.2%)	84 (2.9%)	1,663 (5.3%)	1,747 (5.1%)
**Type of family**						
Children at home	244,875 (54.6%)	983,221 (33.0%)	1,228,096 (35.8%)	909 (31.2%)	6,234 (19.9%)	7,143 (20.9%)
No children at home	203,423 (45.4%)	2,000,634 (67.0%)	2,204,057 (64.2%)	2,000 (68.8%)	25,059 (80.1%)	27,059 (79.1%)
**Education** ^1^						
Compulsory	114,638 (25.8%)	870,007 (32.3%)	984,645 (31.4%)	1,149 (39.5%)	13,899 (44.4%)	15,048 (44.0%)
Secondary	208,856 (47.0%)	1,138,523 (42.3%)	1,347,379 (43.0%)	920 (31.6%)	9,111 (29.1%)	10,031 (29.3%)
University	120,575 (27.2%)	684,072 (25.4%)	804,647 (25.7%)	840 (28.9%)	8,283 (26.5%)	9,123 (26.7%)
**Income quintile** ^2^						
1 (lowest quintile)	97,492 (21.8%)	590,760 (19.8%)	688,252 (20.0%)	—	—	—
2	93,336 (20.8%)	593,387 (19.9%)	686,723 (20.0%)	—	—	—
3	90,760 (20.2%)	594,863 (19.9%)	685,623 (20.0%)	—	—	—
4	85,743 (19.1%)	600,078 (20.1%)	685,821 (20.0%)	—	—	—
5 (highest quintile)	80,967 (18.1%)	604,767 (20.3%)	685,734 (20.0%)	—	—	—
**Country of birth**						
Sweden	418,344 (93.3%)	2,666,384 (89.4%)	3,084,728 (89.9%)	2,909 (100%)	31,293 (100%)	34,202 (100%)
Other Nordic countries^3^	18,421 (4.1%)	150,397 (5.0%)	168,818 (4.9%)	0	0	0
Non-Nordic countries	11,533 (2.6%)	167,074 (5.6%)	178,607 (5.2%)	0	0	0
**Population density - median (interquartile range) inhabitant per square kilometre**						
	56.5 (20.9–123.1)	74.2 (26.5–341.9)	72.5 (26.4–248.2)	45.0 (19.6–107.7)	68.1 (26.3–153.9)	64.2 (25.6–149.9)
**Region of Residence**						
Svealand	158,532 (35.4%)	1,146,703 (38.4)	1,305,235 (38.0)	957 (32.9)	10,949 (35.0)	11,906 (34.8)
Götaland	216,373 (48.2%)	1,429,956 (47.9)	1,646,329 (48.0)	1,396 (47.8)	15,808 (50.5)	17,198 (50.3)
Norrland	73,393 (16.4%)	407,196 (13.7)	480,589 (14.0)	556 (19.1)	4,542 (14.5)	5,098 (14.9)

Numbers and % of the respective cohort are reported unless stated otherwise. ^1^Characteristics of n = 295,482 individuals who had missing values were: 98.6% non-dog owners; 91.3% Swedish-born; median population density 69.3 per km2 (interquartile range, 25.5–341.9); median age 77.5 years (interquartile range, 76.5–79.5); 13.6% resident in Norrland, 49.3% resident in Götaland, 37.1% resident in Svealand; ^2^Information on income not available for the SALT sub-study in the Swedish Twin Register; ^3^Other Nordic countries: Norway, Denmark, Iceland, Finland, the territories of the Åland Islands and the Faroe Islands. ^4^For descriptive purposes, dog owners in this table are individuals who had a registered dog at any time point during the study period.

In age and sex-adjusted analysis, dog ownership was inversely associated with risk of acute myocardial infarction, ischemic stroke, heart failure, and composite CVD (HRs 0.93–0.94; Table 2). After multivariable adjustment, associations with CVD outcomes were attenuated (HRs 0.97–1.01), but remained significant in acute myocardial infarction, HR 0.97 (95% CI 0.95–0.99). Dog ownership was inversely associated with cardiovascular mortality (HR 0.77, 95% CI, 0.73–0.80), and all-cause mortality (adjusted HR 0.80, 95% CI, 0.79–0.82; Table 2). Additional adjustment for education and socioeconomic index in those with enough information available (n = 3,136,671 and n = 1,660,140, respectively) did not affect the results compared to non-adjusted models (Supplementary Tables 4 and 5).

**Table 2:** Hazard ratios (HR) and confidence intervals (CI) examining associations between dog ownership and CVD outcomes in the National Cohort (n = 3,432,153) using Cox proportional hazards regression with attained age as time-scale.

Cardiovascular disease	Number of events	Person-years at risk	Crude^1^ HR (95% CI)	Adjusted^2^ HR (95% CI)
Acute Myocardial Infarction	172,999	37,773,460	0.93 (0.92–0.96)	0.97 (0.95–0.99)
Ischemic Stroke	136,305	37,866,672	0.94 (0.92–0.96)	0.98 (0.96–1.01)
Hemorrhagic Stroke	41,286	38,274,834	0.97 (0.93–1.01)	1.02 (0.98–1.07)
Heart failure	107,843	38,089,903	0.93 (0.90–0.96)	1.01 (0.98–1.05)
Composite CVD^3^	399,600	36,910,720	0.94 (0.93–0.96)	0.99 (0.98–1.01)
CVD mortality^4^	76,106	38,408,267	0.68 (0.65–0.71)	0.77 (0.73–0.80)
All–Cause mortality	502,896	38,408,267	0.72 (0.71–0.73)	0.80 (0.79–0.82)

^1^Adjusted for sex. ^2^Adjusted for sex, marital status, presence of children in the home, population density, area of residence, region of birth, income and latitude. ^3^Composite CVD comprises all incident (fatal and non-fatal) ischemic stroke, myocardial infarction, hemorrhagic stroke and heart failure cases. ^4^Representing death from ischemic strokes, myocardial infarction, hemorrhagic stroke and heart failure.

**Figure 1:**
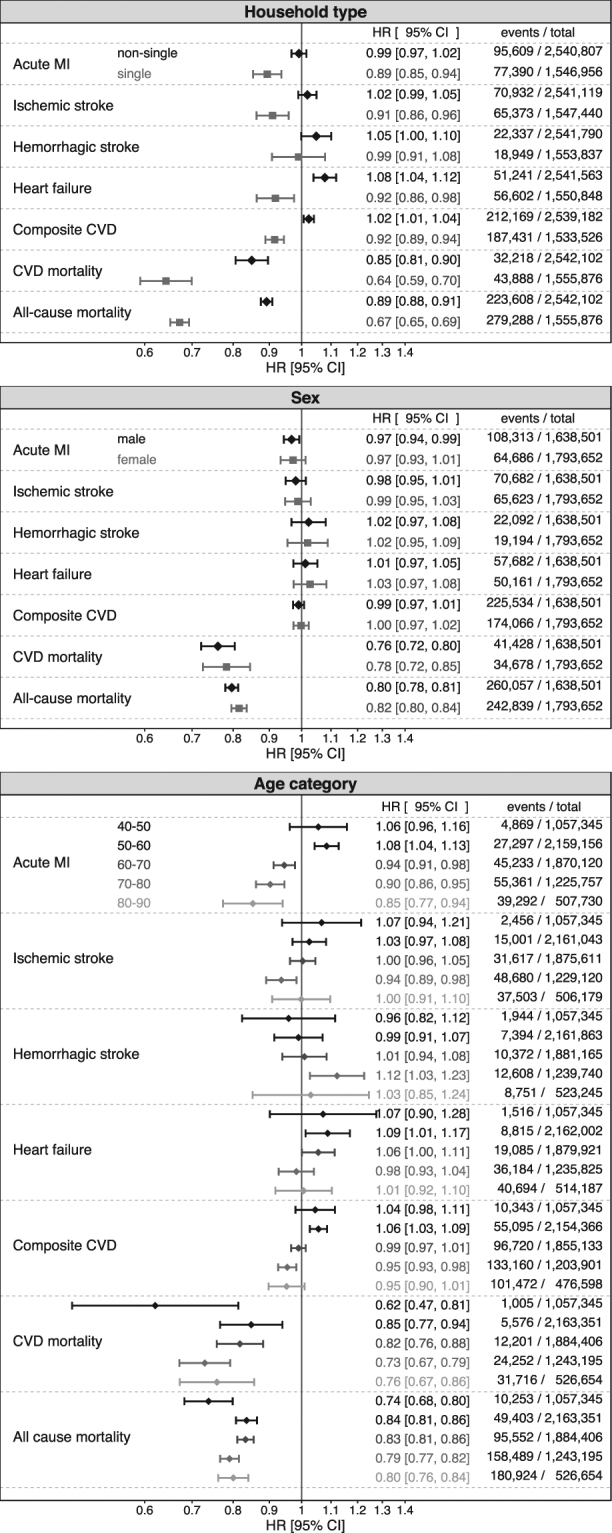
Hazard ratios (HR) and confidence intervals (CI) of the associations between dog ownership and CVD outcomes in the National cohort stratified by household type, sex, and age using Cox proportional hazards regression with attained age as time-scale.

Stratified analysis showed effect measure modification of household type for all outcomes except haemorrhagic stroke, with lower multivariable adjusted HRs found in single households (Fig. 1, upper panel). There seemed to be no effect measure modification by sex (Fig. 1, middle panel). Age seemed to modify the association between dog ownership and myocardial infarction, with lower HRs in older age groups (Fig. 1, lower panel).

In the breed group analysis, we found that ownership of a dog from breeds originally bred for hunting (including terriers, retrievers, scent hounds and related dogs) was associated with a lower risk of CVD (Table 3). Ownership of a mixed-breed dog was associated with higher risk of CVD, HR 1.13 (95% CI, 1.09–1.17). The HR for all-cause mortality was <1 for all breed groups, with pointing dogs associated with the lowest estimate (HR 0.60, 95% CI, 0.53–0.68) and mixed-breed dogs with estimates closest to one (HR 0.98, 95% CI, 0.94–1.01).

**Table 3:** Hazard ratios (HR) and 95% confidence intervals (CI) for associations between dog breed groups and CVD outcomes in the National cohort using Cox proportional hazards regression with attained age as time-scale.

Breed Groups	Composite Cardiovascular Disease^1^		All-Cause Mortality	
	Crude HR^2^	Adjusted HR^3^	Crude HR^2^	Adjusted HR^3^
Sheep and cattle dogs	0.99 (0.95–1.03)	1.02 (0.98–1.07)	0.76 (0.73–0.80)	0.84 (0.80–0.88)
Pinscher and schnauzer dogs	0.91 (0.87–0.95)	0.97 (0.93–1.02)	0.68 (0.64–0.72)	0.78 (0.74–0.82)
Terriers	0.88 (0.84–0.92)	0.95 (0.91–0.99)	0.70 (0.67–0.74)	0.81 (0.78–0.86)
Dachshunds	0.88 (0.83–0.93)	0.94 (0.89–1.00)	0.67 (0.63–0.71)	0.76 (0.72–0.81)
Spitz and primitive types	0.97 (0.92–1.02)	0.98 (0.94–1.03)	0.68 (0.65–0.72)	0.72 (0.68–0.76)
Scent hounds and related dogs	0.90 (0.86–0.95)	0.93 (0.88–0.97)	0.58 (0.55–0.62)	0.63 (0.60–0.67)
Pointing dogs	0.84 (0.77–0.93)	0.90 (0.82–1.00)	0.52 (0.46–0.59)	0.60 (0.53–0.68)
Retrievers	0.83 (0.80–0.86)	0.90 (0.87–0.94)	0.63 (0.60–0.66)	0.74 (0.71–0.77)
Companion and toy dogs	1.00 (0.96–1.03)	1.04 (1.01–1.08)	0.77 (0.74–0.80)	0.85 (0.82–0.89)
Sight hounds	0.97 (0.83–1.14)	1.02 (0.87–1.18)	0.76 (0.64–0.90)	0.83 (0.70–0.99)
Mixed Pedigree^4^	1.12 (1.08–1.16)	1.13 (1.09–1.17)	0.93 (0.90–0.97)	0.98 (0.94–1.01)

^1^Composite CVD comprises all ischemic stroke, myocardial infarction, hemorrhagic stroke and heart failure. ^2^Adjusted for sex. ^3^Adjusted for sex, marital status, presence of children in the home, population density, area of residence, region of birth, income and latitude. ^4^Group comprising all non-pure pedigree dogs.

### Swedish Twin Register

The final dataset included 34,202 individuals (45% men) with a mean age of 57 years. We identified 8.5% as registered dog owners at any time during the 14-year follow-up period. The cohort had similar characteristics as the national cohort, except that fewer individuals lived in single households, probably due to differences in accuracy of measurement (register not complete for individuals cohabiting without common children vs phone survey in which partner information was recorded) (Supplementary Table 6).

**Figure 2:**
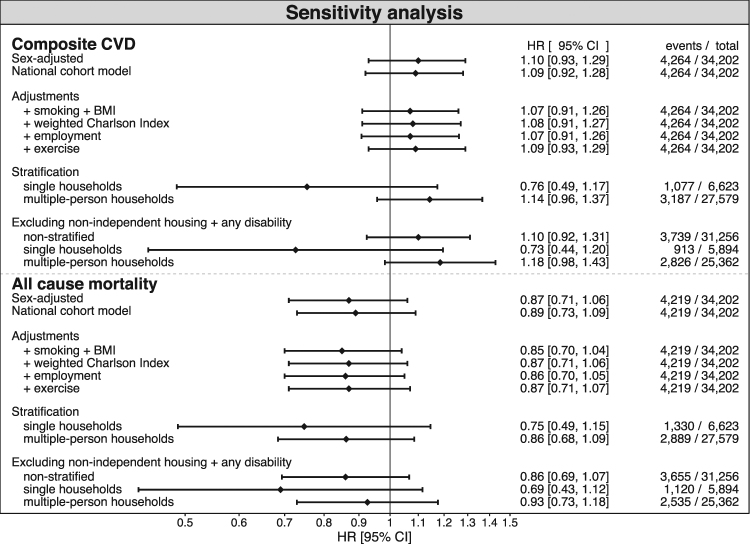
Association of dog ownership with cardiovascular disease and all-cause mortality in the Swedish Twin Register following adjustments and exclusions in sensitivity analysis using Cox proportional hazards regression with attained age as time-scale. The “National cohort model” was adjusted for sex, marital status, type of family, area of residence, education level, population density and occupation level.

Multivariable-adjusted estimates for composite CVD, HR 1.09 (95% CI, 0.92–1.28), and all-cause mortality, HR 0.89 (95% CI 0.73–1.09) overlapped with estimates from the national cohort, but the estimate for CVD was in the opposite direction. The serial addition of the different lifestyle covariates to the model only slightly altered the estimates (Fig. 2). Overall, the results from the Twin Register did not demonstrate any difference in risk of CVD or mortality between dog owners and non-owners but remained robust to a range of different modifications explored in sensitivity analyses, including the exclusion of individuals with disabilities or unable to care for themselves (Fig. 2).

## Discussion

In this register-based nationwide prospective study including 3,432,153 individuals, dog ownership was associated with a lower risk of incident cardiovascular disease in single-person households and with lower cardiovascular and all-cause mortality in the general population. Ownership of hunting dog breeds was associated with a decreased risk of CVD, and ownership of all purebred breeds were associated with a lower risk of all-cause mortality. Although further investigation in the Twin cohort did not show any association between dog ownership and CVD and mortality likely due to the smaller sample size, additional adjustment for detailed lifestyle and socioeconomic factors only marginally altered these estimates.

A recent Norwegian prospective cohort study within a subset of the HUNT study (n = 28,746 in adjusted analysis, 4,233 events) showed no difference in all-cause mortality between dog owners and non-owners (adjusted HR 1.00, 95% CI 0.91–1.09)^29^. Adjustments were similar as in our study, but stratification by family type was not investigated, which would have been interesting to compare to our results, as the single household stratum was where the largest protective effect sizes were observed. The differences in results from our study could be due to differences in the population studied (largely rural in the HUNT study), difference in the study design (cohort vs register-based), measurement of dog-ownership (questionnaire vs register) and time of exposure definition (baseline vs time-updated) or pure chance. Three other previous studies conducted within the Second and Third National Health and Nutrition Examination Survey (NHANES II-III) of 4,435 (44% dog owners), 5,903 (11% dog owners) and 3,964 (22% dog owners) individuals, respectively, reported no effect of dog ownership on all-cause mortality (adjusted HRs 1.00, 95% CI, 0.85–1.20; 1.17, 95% CI, 0.94–1.46; and 0.82, 95% CI, 0.51–1.34, respectively)^30–32^. Whilst statistical methods were similar to our study, these studies suffered from a high degree of incomplete follow-up (~50%) and small sample sizes, which do not apply to the present study based on comprehensive registers of the total population. Further, three smaller unrelated studies in the United States support an association of dog ownership with lower mortality after cardiovascular events^14,16,33^.

Our sample size is hundredfold larger than the largest previously reported study^29^ and allows for more precise effect estimates and analyses of different CVD outcomes and in different subgroups. Using updated exposure information on dog ownership, we reduce the influence and analyses of misclassification. Cumulative dog ownership in the national cohort was similar to the 12.9% estimate in a cross-sectional 2012 survey and somewhat lower in the Swedish Twin Registry cohort since we were not able to take partners’ registration of dog ownership into account.

Our observational study cannot provide evidence for a causal effect of dog ownership on cardiovascular disease or mortality. Although careful attention was paid to adjusting for potential confounders in a set of sensitivity analyses, it is still possible that personal characteristics that we did not have information about affect the choice of not only acquiring a dog, but also the breed and the risk of CVD. Such residual confounding in the present study may be indicated by the opposite direction of the association with CVD in mixed-breed dogs.

There might be direct effects of dog ownership on health outcomes. One mechanism by which dog ownership could reduce CVD risk and mortality is by alleviating psychosocial stress factors, such as social isolation, depression and loneliness - all reportedly lower in dog owners^3–5,34^. These factors have been linked to increased risk of coronary heart disease, cardiovascular death and all-cause mortality^35,36^. Dog ownership has also been associated with elevated parasympathetic and diminished sympathetic nervous system activity^37^, lower reactivity to stress, and faster recovery of blood pressure following stressful activity^38^. Apart from the social support, it has consistently been shown that dog owners achieve more physical activity and spend more time engaged in outdoor activities^4^. We found that individuals in single households benefitted most from dog ownership regarding protection from CVD. Here, we defined a single household based on both marital status and the presence of children in the household. A study on the psychological effects of dog ownership suggested that ownership benefits single persons more than married individuals^3^ Moreover, single dog-owners were shown to walk their dog more often than individuals in multiple-person households^39^, and in general, it is plausible that not all members of a multiple-person household interact with the dog as much as a single owner. In both multiple- and single-person household strata, we found lower hazard ratios for dog ownership on all-cause mortality and cardiovascular mortality than on incident CVD. This discrepancy may be explained by less severe disease at hospital presentation in dog-owners, similar to the effect described in physically active persons^40^. Alternatively, owning a dog may improve rehabilitation after an incident disease event by acting as motivation and support to mobilize for walks again.

To our knowledge, our prospective nationwide study is by far the largest investigation of associations of dog ownership with human health reported to date. The comprehensiveness of the Swedish population register system gave us access to a rich set of possible confounders and outcomes, allowed for accurate censoring and low numbers of missing data or lost participants. Our results are based on the Swedish population and likely generalizable to other European populations with similar demographics and cultural practices regarding dog ownership. We found statistically robust estimates that were consistent, although with lower precision, after adjustment in the smaller twin cohort for lifestyle factors such as smoking, obesity, physical impediment, assisted living and comorbidity.

Some limitations apply to our study. As earlier mentioned, there is a risk of unmeasured confounding, especially from personal characteristics affecting the choice of not only acquiring a dog, but also lifestyle and the risk of CVD and death. Further, although we excluded individuals with a history of CVD at baseline and adjusted for comorbidities in sensitivity analyses, there remains a risk of reverse causation, in that individuals with disabilities or comorbidity may be less likely to own a dog, and may be at higher risk for CVD and death. Although registration of dog ownership is mandatory in Sweden, not all dogs are registered, and we noted that the number of dogs in the Board of Agriculture Register increased during the study period. Some dog owners might be misclassified as non-owners in the beginning of the study period, which could have introduced bias away from the null if misclassification was associated with cardio-protective factors. Likelihood of dog registration could also be linked to factors like personality that we were not able to control for.

Taken together, we believe our longitudinal population-wide design provides the most robust evidence so far of a link between dog ownership and health outcomes, although bias from reverse causation, misclassification and confounding cannot be excluded.

## Conclusion

In conclusion, in a nationwide population based study with 12 years of follow-up, we show that dog ownership is associated with a lower risk of cardiovascular disease in single households and with a reduced risk of cardiovascular and all-cause death in the general population.

### Infrastructure support

Support by NBIS (National Bioinformatics Infrastructure Sweden) for the use of the Mosler system for sensitive data is gratefully acknowledged. There was no compensation received for this assistance.

We would also like to acknowledge the Swedish Kennel Club and the National Board of Agriculture for granting access to the dog registers. They were not involved in any part of the study design, analysis, data interpretation, manuscript preparation or approval.

### Scientific support

We acknowledge the assistance provided by Malin Ericsson of the Department of Medical Epidemiology and Biostatistics, Karolinska Institutet, Sweden in defining the socioeconomic variables from the Swedish Twin Registry.

We acknowledge the assistance provided by Dr Erik Bihagen of the Swedish Institute for Social Research in defining the socioeconomic variables in the Register of the Total Population.

We acknowledge Dr Mark S. Clements of the Department of Medical Epidemiology and Biostatistics, Karolinska Institutet, Sweden for assistance with the statistical analysis.

## Electronic supplementary material

Supplementary Material
