# Supplementary material for: Dog ownership and the risk of cardiovascular disease and death – a nationwide cohort study
Source: Sci Rep. 2017 Nov 17;7:15821. doi: 10.1038/s41598-017-16118-6 (PMC5693989; doi:10.1038/s41598-017-16118-6)
Supplement: Supplementary file 1 — Supplementary Material [file 41598_2017_16118_MOESM1_ESM.pdf]

Supplementary Material

Dog ownership and the risk of cardiovascular disease and death – a nationwide cohort study

Mwenya Mubanga, MBChB, MPH[1]; Liisa Byberg, PhD[2]; Christoph Nowak, BM, BCh, PhD[1,3]; Agneta Egenvall, VMD, PhD[4]; Patrik K Magnusson, PhD[5]; Erik Ingelsson, MD, PhD[1,6]; Tove Fall, VMD, PhD[1*]

1. Department of Medical Sciences, Molecular Epidemiology and Science for Life Laboratory, Uppsala University, Uppsala, Sweden.
2. Department of Surgical Sciences, Orthopedics, Uppsala University, Uppsala, Sweden.
3. Department of Neurobiology, Care Sciences and Society, Karolinska Institutet, Huddinge, Sweden.
4. Department of Clinical Sciences, Division of Ruminant Medicine and Veterinary Epidemiology, Swedish University of Agricultural Sciences, Uppsala, Sweden.
5. Department of Medical Epidemiology and Biostatistics, Karolinska Institutet, Stockholm, Sweden.
6. Department of Medicine, Division of Cardiovascular Medicine, Stanford University School of Medicine, Stanford, CA, USA.

*Corresponding author: Tove Fall, VMD, PhD, tove.fall@medsci.uu.se

Content

Supplementary Methods

Record Linkage

Marital status definition

Charlson comorbidity index

Social stratification and occupational classifications

Dog breed classification

Supplementary Table 1-6

Supplementary Figure 1-3

References

Supplementary Methods

Record linkage

Several databases were used in the analysis of the information provided in this manuscript.

Since 1967, it has been a statutory requirement that every Swedish resident be registered in the Register of the Total Population and assigned a unique identifier, the personal identity number (PIN) [1].

The PIN made it possible to link information between the Register of the Total Population (RTP) and the registers of the Longitudinal Integration Database for Health Insurance and Labor Market Studies for socioeconomic indicators, the National Patient Registers for health outcomes and the Cause of Death Register for vital status. Additionally, exposure to dogs was determined by linkage to the Swedish Kennel Club and Swedish Board of Agriculture dog registers. Individual-level record-linkage was conducted by Statistics Sweden and the National Board of Health and Welfare.

In the second part of our study, replication was done in the Swedish Twin Register (STR). This is a longitudinal database established in the late 1950's. It contains information on all twins born in Sweden from 1886 onwards and has been used in several studies [2, 3]. The Screening Across Lifespan Twin sub-study was initiated in 1998 in order to screen all twins born before 1958, regardless of the gender composition or vital status of the pair. It was based on a computer-assisted telephone interview including a number of items asked to all twins regarding different lifestyle habits, diseases and symptoms. Efforts were made to interview members of a pair within a month of each other to minimize the risk of biasing the results by differential age effects [4].

Similar to the National cohort, participants from the STR were linked to the National Patient Registers for health outcomes, the Cause of Death Register and, exposure to dogs was determined by linkage to the Swedish Kennel Club and Swedish Board of Agriculture dog registers. There was no partner information available in the Twin Register.

Marital Status

Study participants were assigned to the "married/cohabiting" category if they were registered as either married, living in a registered (same-sex) partnership or being a cohabiting couple with children living in the home or elsewhere regardless of the age of the children. Other categories – single, divorced or widowed, were extracted from the civil status as recorded in the Register of The Population.

Charlson Comorbidity Index

Diagnosis codes (ICD codes 9–10), were extracted from the National Patient Register for the period from January 1st, 1996 to December 31st, 2000 in the Twin cohort. This was done to calculate the number of Charlson comorbidities for each person prior to the study onset. The Charlson comorbidity index predicts the 10 year mortality for a patient who may have a range of comorbid conditions (up to 17 diseases) [5, 6]. This was done to investigate potential bias from recent disease on dog ownership. The diagnoses used to define the Charlson comorbidity score are shown in **Supplementary Table 2**. To note is that in the present study, all individuals with prevalent CVD were excluded from the population.

Social stratification and occupational classifications

Sensitivity analyses testing for residual confounding was conducted in the National cohort by adjusting for education, occupational status and income. The analyses were restricted to the population for whom education, income, and occupation information was available. Based on international classification, we adjusted for an occupational-based variable (ESeC – European Socio-economic Classification) which is an occupationally based standard classification [7]. We also adjusted for a socio-economic index variable (ISEI – International Socio-Economic Index) which scales occupations by the average level of education and job earnings of job holders) [8].

Dog breed classification

The breed classification system used was based on the classification system used by the Swedish Kennel Club as derived from the Nordic Kennel Union based on the Fédération Cynologique Internationale system [9] (**Supplementary Table 1**).

Supplementary Table 1. Description of Breed Classification of the 339 breeds included in the study based on the Nordic Kennel Union Classification

Group Number	Breed Groups	Breed Code
1	Sheep and cattle dogs	Sheep dogs (Australian, Belgian, Catalan, German, Picardy, Polish, Portuguese, Pyrenean, Shetland, Old English); Shepherd dogs (Belgian, Bergamasco, Croatian, Dutch, German, Majorca, Polish, Romanian, South Russian); Collie (Bearded, Border, Rough, Smooth); Bouvier des Flandres, Beauceron, Briard, Chodsky Pes, Czechoslovakian Wolfdog, Komondor, Kuvasz, Mudi, Lancashire Heeler, Schipperke, Puli, Pumi, Slovakian Chuvach, Welsh Corgie, Australian kelpie, Working kelpie
2	Pinscher and schnauzer dogs	Pincher (Affenpinscher, Austrian, Dobermann, German, Miniature); Schnauzer (Giant, Miniature); Mountain Dog (Appenzeller, Bernese, Caucasian Shepherd, Entlebuch, Great Swiss, Karst, Landseer, Newfoundland, Pyrenean, Serra da Estrela, St Bernard, Uruguayan Cimarron, Yugoslavian Shepherd); Molossian (Aidi, Anatolian Shepherd, Boxer, Bull Mastiff, Broholmer, Cane Corso, Dogo Argentino, Danish-Swedish Farm dog, Dogo Canario, Dogue de Bordeux, English Bulldog, Great Dane, Hovawart, Majorca Mastiff, Mastiff, Neapolitano Mastiff, Pyrenese Mastiff, Rafeiro of Alentejo, Spanish Water Dog, Shar Pei, Tosa); Central Asia Shepherd Dog, Russian Black Terrier
3	Terriers	Airedale, American Staffordshire, Australian, Bedlington, Border, Brazilian, Bull, Cairn, Cesky, Dandie Dinmont, English Toy, Fox, German Hunting, Irish Glen of Imaal, Irish Softcoated Wheaton, Irish, Jack Russel, Kerry Blue, Lakeland, Manchester, Miniature Bull, Norfolk, Norwich, Parson Russell, Sealyham, Australian Silky, Skye, Tenterfield, Welsh, West Highland White, Yorkshire
4	Dachshunds	Miniature, Standard, Kaninchen
5	Spitz and primitive types	Alaskan Malamute, American Akita, Canaan dog, Canarian Warren, Chow Chow, Cirneco dell'Etna, East Siberian Laika, Eurasian, Finnish Lapphund, Finnish Spitz, German Spitz, Greenland dog, Hokkaido, Halleforshund, Icelandic Sheepdog, Japanese Akita, Japanese Spitz, Karelian Beardog, Keeshond, Korea Jindo, Laponian Herder Pharaoh Hound, Mexican Hairless dog, Norwegian Buhund, Norwegian Lundehund, Norwegian Elkhound, Peruvian Hairless dog, Ibizan Hound, Pomeranian, Russian European Laika, Samoyed, Shiba, Siberian Husky, Swedish Elkhound, Swedish Lapphund, Swedish White Elkhound, Swedish Vallhund, Thai Bangkaew, Thai Ridgeback, Volpino italiano, West Siberian Laika
6	Scent hounds and related dogs	Alpine Dachsbracke, American Foxhound, Basset Artesian Nomand, Basset Bleu de Gascogne, Basset fauve de Bretagne, Basset Hound, Bavarian Mountain Scent hound, Beagle, Black and Tan Coonhound, Bloodhound, Bluetick Coonhound, Bosnian Coarse-haired hound, Dalmatian, Drever, Dunker Hound, Fawn Brittany Griffon, Finnish Hound, Foxhound, German Hound, Grand Basset Griffon Vendeen, Grand Griffon Vendeen, Griffon Nivernais, Halden Hound, Hamilton Hound, Hygen Hound, Istrian Short-haired hound, Otterhound, Petit Basset Griffon Vendeen, Plott, Polish hunting dog, Porcelain, Posavaz Hound, Rhodesian Ridgeback, Russian Hound, Russian Spotted hound, Small Blue Gascony Hound, Spanish Hound, Schiller Hound, Swiss Hound, Serbian Hound, Slovakian Hound, Småland Hound
7	Pointing dogs	Blue Picardy Spaniel, Bracco Italiano, French Pointing, Brittany, Bohemian wire-haired, Drentse Patridge, English Setter, French Spaniel, Old Danish Pointer, Gordon Setter, French wire-haired Korthals Pointing Griffon, Münsterländer, Irish Red Setter, German Short/Wire-haired pointing dog, Portuguese Pointing dog, Pointer, Pudelpointer, Slovakian Wire-haired Pointing dog, Italian Spinone, Stabyhound, Hungarian Vizsla wire-/short-haired, Weimaraner short-/long-haired
8	Retrievers	American Cocker Spaniel, Barbet, Chesapeake Bay Retriever, Clumber Spaniel, Cocker Spaniel, Curly Coated Retriever, English Springer Spaniel, Field Spaniel, Flat coated Spaniel, German Spaniel, Golden retriever, Irish Water Spaniel, Labrador Retriever, Lagotto romagnolo, Nederlandse Kooikerhondje, Nova Scotia

		Duck Tolling Retriever, Spanish Water dog, Portuguese Water Dog, Sussex Spaniel, Welsh Springer Spaniel, Wetterhound
9	Companion and toy dogs	Havanese, Bolognese, Boston Terrier, Belgian Griffon, Brussels Griffon, Cavalier King Charles Spaniel, Chihuahua, Chinese Crested, Coton de Tulear, French Bulldog, Japanese Chin, King Charles Spaniel, Kromfohrlander, Lhasa Apso, Lowchen, Maltese, Pug, Papillon, Pekingese, Small Brabant Griffon, Phalene, Prazský krysarík, Poodle, Russian Toy, Shih Tzu, Tibetan Terrier, Tibetan Spaniel
10	Sight hounds	Afghan Hound, Azawakh, Borzoi, Polish Greyhound, Spanish Greyhound, Irish Wolfhound, Italian Greyhound, Hungarian Greyhound, Saluki, Scottish Deerhound, Sloughi, Whippet

Supplementary Table 2. Description of variables derived from the SALT questionnaire study

Covariate	Questionnaire Option	Variable created	Classification and Derivative from questionnaire
**Marital status**	What is your civil status?	Married<br>Single<br>Divorced<br>Widowed	Married, cohabiting<br>Living alone<br>Divorced, separated, living apart<br>Widow/ widower
**Type of family**	Living with children <18 years	Yes/ No	Yes /No
**Education level**	Highest years of education completed	Primary education or less<br>Secondary education<br>Tertiary education or more	9 years or less of education<br>10 to 12 years of education<br>More than 12 years of education
**Employment status**	Employment status	Employed<br><br><br>Retired<br>Retired for disability or illness<br>Unemployed	Fully employed, part time employment, owns company, on leave from work, study leave or on military service<br>Pensioner, prematurely retired, partly retired<br>Retired for injury<br>Unemployed, housewife/man
**Socioeconomic index**	Socioeconomic occupation level	Level 1<br>Level 2<br>Level 3<br><br>Level 4<br>Level 5	Unskilled Employees<br>Lower skilled, non-manual workers<br>Self-employed excluding independent workers<br>Intermediate non-manual employees<br>Highest tier non-manual employees
**Tobacco Use**	Have you ever smoked or used snuff	Never smoked<br><br>Former smoker<br><br>Current smoker	No not even tried it, yes but only tried it, smoked now and then (like at parties),<br>Smoked regularly, snuffed regularly, smoke now and then (like at parties)<br>Smoke regularly, smoke at parties, snuff now and then, snuff regularly
**Any movement impairment**	Do you have any physical handicap	Yes/no	Yes/ No
**Function**	Do you need assistance with personal care/ shopping,/cooking/mobility/	Yes/No	Yes /No
**Exercise**	How much do you exercise; what fits your annual exercise pattern	Less than average<br><br>Average<br>More than average	Almost no exercise, light exercise, much less exercise than average, less than average<br>Regular medium exercise, average amount of exercise<br>Hard physical exercise, more exercise than average, much more exercise than normal, maximum amount of exercise

Supplementary Table 3. Charlson comorbidity index components and weights derived from primary and secondary diagnoses

Weights	Conditions	ICD-9 codes[1]	ICD-10 codes[1]
1	Myocardial infarction	410,412	I21, I22, I25
	Congestive heart failure	425E, 425F, 425G, 425H, 425W, 425X, 428	I099, I110, I130, I132, I255, I420, I425, I426, I427- I429, P230
	Peripheral vascular disease	093A, 437D, 443B, 443W, 443X, 447B, 557B, 557X, V43E, 440, 441	I731, I738, I739, I771, I790, I792, K551, K558, K559, Z958 Z959
	Dementia	290, 294B , 331C	F051, G311, F00, F01, F02, F03, G30
	Cerebrovascular disease	430-438	H340, G45, G46, I60- I69
	Chronic pulmonary disease	491-505	I278, I279, J684, J701, J703, J40-J47, J60-J67
	Connective tissue disease	446F, 710A, 710B, 710C, 710D, 710E, 714A, 714B, 714C, 714W, 725	M05, M06, M32- M34, M315, M351, M353, M360
	Ulcer disease	531-534	K25-K28
	Mild liver disease	570, 571, 070C, 070D, 070E , 070F, 070G , 070X, 573D, 573E, 573W, 573X, V42H	K700-K703, K709, K713-K715, K717, K760, K762-K764, K768, K769, Z944
	Diabetes(without complications)	250A, 250B, 250C, 250H, 250X	E100, E101, E106, E108, E109, E110, E111, E116, E118- E121, E126, E128, E129-E131, E136, E138- E141, E146, E148, E149
2	Hemiplegia	342, 433, 334B, 344A, 344B, 344C, 344D, 344E, 344F, 344G, 344X	G041, G114, G801, G802, G830-G832, G834, G839
	Moderate or severe renal disease	403A , 403B, 403X, 404A, 404B, 404X, 583A, 583B, 583C, 583E , 583G , 583H, 588A, V42A, V45B, 582, 585, 586, V56	I120, I131, N032-N037, N052 - N057, N250, Z490-Z492, Z940, Z992
	Diabetes (with chronic complications)	250D, 250E, 250F, 250G	E102-E105, E107, E112- E115, E117, E122, E123-E125, E127, E132-E135, E137, E142-E145, E147
	Any tumor	14-16, 18, 190-195, 170-172, 174-179, 238G	C0, C1, C6, C20 C21-C26 , C30-C34, C37-C39, C40, C41, C43-C58 , C70-C76
	Leukemia	204-208	C90 - C97
	Lymphoma	200-203	C81 - C85, C88
3	Moderate or severe liver disease	456A, 456B, 456C, 572C, 572D, 572E, 572W	I850, I859, I864, I982, K704, K711, K721, K729, K765-K767
6	Metastatic solid tumor	196-199	C77-C80
	Acquired Immune Deficiency Syndrome	042, 043, 044	B20- B22 , B24

1. The ICD-9 and ICD-10 codes are modified for the National Patient Registers to be compatible with the Swedish Disease Classification system.

Supplementary Table 4. Hazard ratios (HR) and confidence intervals (CI) examining associations between dog ownership and CVD outcomes in the National Cohort with additional adjustment for education (n=3,136,671) in a subset of the population born >1925 with education data available

**Cardiovascular disease**	**Number of events**	**Person-years at risk**	**Crude[1] HR (95% CI)**	**Adjusted[2] HR (95% CI)**	**Fully adjusted HR with education[3] (95% CI)**
Acute Myocardial Infarction	133,723	35,259,660	0.95 (0.93-0.97)	0.98 (0.96-0.99)	0.98 (0.96-1.01)
Ischemic Stroke	100,007	35,368,780	0.94 (0.92-0.97)	0.99 (0.96-1.02)	0.99 (0.97-1.02)
Hemorrhagic Stroke	33,054	35,676,189	0.97 (0.93-1.01)	1.02 (0.98-1.07)	1.03 (0.98-1.07)
Heart failure	70,883	35,555,936	0.93 (0.90-0.96)	1.02 (0.99-1.06)	1.03 (0.99-1.06)
Composite CVD[4]	299,498	34,583,324	0.95 (0.94-0.97)	1.00 (0.99-1.02)	1.00 (0.98-1.02)
CVD mortality[5]	45,648	35,791,138	0.68 (0.65-0.72)	0.78 (0.75-0.82)	0.79 (0.75-0.83)
All-Cause mortality	334,010	35,791,138	0.72 (0.71-0.74)	0.82 (0.80-0.83)	0.82 (0.81-0.84)

1. Adjusted for sex and age
2. Adjusted for sex, age, marital status, presence of children in the home, population density, area of residence, region of birth, income and latitude
3. Adjusted for sex, age, marital status, presence of children in the home, population density, area of residence, region of birth, income, latitude and education
4. Composite CVD comprises all ischemic stroke, myocardial infarction, hemorrhagic stroke and heart failure.
5. Representing death from ischemic strokes, myocardial infarction, hemorrhagic stroke and heart failure.

Supplementary Table 5. Hazard ratios (HR) and confidence intervals (CI) examining associations between dog ownership and CVD outcomes in the National Cohort with additional adjustment for socioeconomic index (n=1,660,140) in a subset of the population with profession information available

Cardiovascular disease	Number of events	Person-years at risk	Main Model[1] HR (95% CI)	ISEI-Adjusted[2] HR (95% CI)	ESeC-Adjusted[3] (95% CI)
Acute Myocardial Infarction	63,325	19,397,364	1.00 (0.97-1.04)	1.00 (0.97-1.04)	1.00 (0.97-1.04)
Ischemic Stroke	50,728	19,443,313	0.99 (0.96-1.03)	0.99 (0.96-1.03)	0.99 (0.96-1.03)
Hemorrhagic Stroke	16,246	19,602,018	1.04 (0.98-1.10)	1.04 (0.98-1.11)	1.04 (0.98-1.11)
Heart failure	33,830	19,547,382	1.07 (1.02-1.12)	1.06 (1.01-1.12)	1.07 (1.02-1.12)
Composite CVD[4]	145,880	19,050,912	1.02 (1.00-1.05)	1.02 (1.00-1.04)	1.02 (1.00-1.05)
CVD mortality[5]	18,437	19,662,284	0.91 (0.84-0.97)	0.91 (0.84-0.97)	0.91 (0.85-0.98)
All-Cause mortality	141,249	19,662,284	0.94 (0.92-0.96)	0.94 (0.91-0.96)	0.94 (0.92-0.97)

1. Main model – adjusted for sex, age, marital status, presence of children in the home, population density, area of residence, region of birth, latitude, income and education
2. ISEI-adjusted – M\main model + ISEI (where ISEI is a socio-economic index of occupational status which scales occupations by the average level of education and average earnings of job holders)[8]
3. ESeC-adjusted – main model + ESeC (where European Socio-economic Classification is an occupationally based classification)[7]
4. Composite CVD comprises all ischemic stroke, myocardial infarction, hemorrhagic stroke and heart failure.
5. Representing death from ischemic strokes, myocardial infarction, hemorrhagic stroke and heart failure.

Supplementary Table 6. Additional baseline characteristics of 34,202 Swedish adults in the Swedish Twin Register without cardiovascular disease on 1 January 2001

	Twin cohort		
	Dog owners n=2,909 (8.5%)	Non-dog owners n=31,293 (91.5%)	All n=34,202 (100%)
**Body mass index[1] -mean ± SD**	25.0 ± 3.6	25.0 ± 3.5	25.0 ± 3.5
**Diabetes**	94 (3.2%)	1,221 (3.9%)	1,315 (3.8%)
**Any movement impairment**	293 (10.1%)	2,358 (7.5%)	2,651 (7.8%)
**Exercise frequency**			
Little or none	644 (22.1%)	8,556 (27.3%)	9,200 (26.9%)
Average	640 (22.0%)	8,066 (25.8%)	8.706 (25.5%)
Above average	1,625 (55.9%)	14,671 (46.9%)	17,620 (47.3%)
**Smoking**			
No history of smoking	838 (28.8%)	12,291 (39.3%)	13,129 (39.0%)
Previous smoker	1,300 (44.7%)	12,249 (39.1%)	13,549 (39.1%)
Current Smoker	771 (26.5%)	6,753 (21.6%)	7,524 (21.9%)
**Type of housing or accommodation**			
Independent	2,908 (100.0%)	31,233 (99.8%)	34,141 (99.8%)
Assisted living[2]	0 (0%)	42 (0.1%)	42 (0.1%)
Other	1 (<0.1%)	18 (<0.1%)	19 (<0.1%)
**Employment status**			
Employed	2,188 (75.2%)	20,273 (64.8%)	22,461 (65.7%)
Retired	259 (8.9%)	7,467 (23.9%)	7,726 (22.6%)
Sick leave or illness	368 (12.7%)	2,692 (8.6%)	3,060 (8.9%)
Unemployed	94 (3.2%)	861 (2.8%)	955 (2.8%)
**Profession[3]**			
Unskilled labor	810 (27.8%)	8,536 (27.3%)	9,346 (27.3%)
Lower non-manual labor	947 (32.6%)	10,752 (34.4%)	11,699 (34.2%)
Self-employed	175 (6.0%)	1,438 (4.6%)	1,613 (4.7%)
Intermediate non-manual labor	641 (22.0%)	6,813 (21.8%)	7,454 (21.8%)
Higher non-manual employee	336 (11.6%)	3,754 (12.0%)	4,090 (12.0%)
**Weighted Charlson Comorbidity index[4]**			
0	2,817 (96.8%)	29,805 (95.2%)	32,622 (95.6%)
1	51 (1.8%)	731 (2.3%)	782 (2.1%)
≥2	41 (1.4%)	757 (2.4%)	798 (2.2%)

1. kg/m2
2. Living in a nursing home, retirement home, group living home or hospital
3. Defined according to Budoki et al [10]
4. Total score, ranging from 0 to 17 with higher scores indicating more severe comorbidity

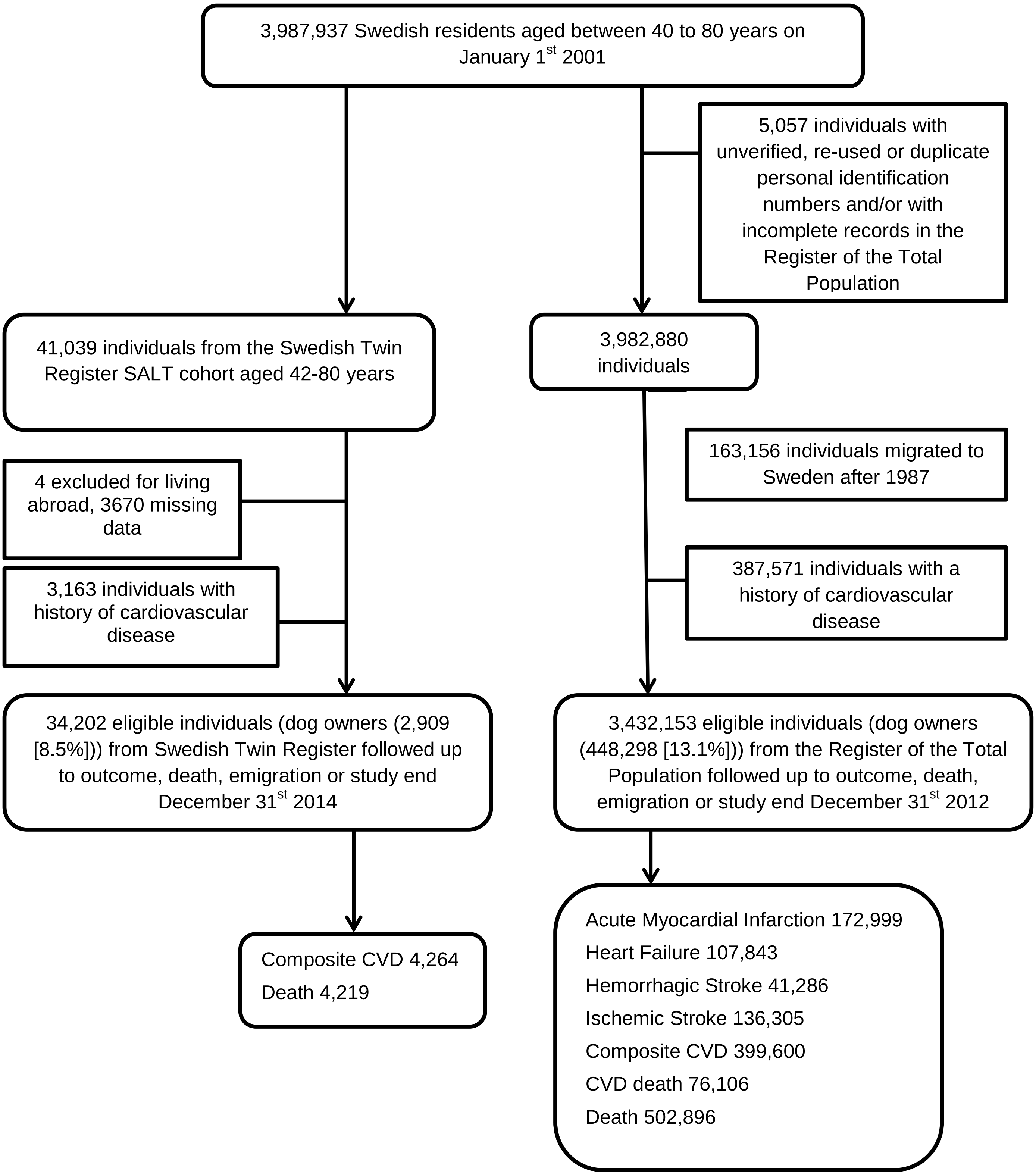

Supplementary Figure 1. Flow chart of the study design

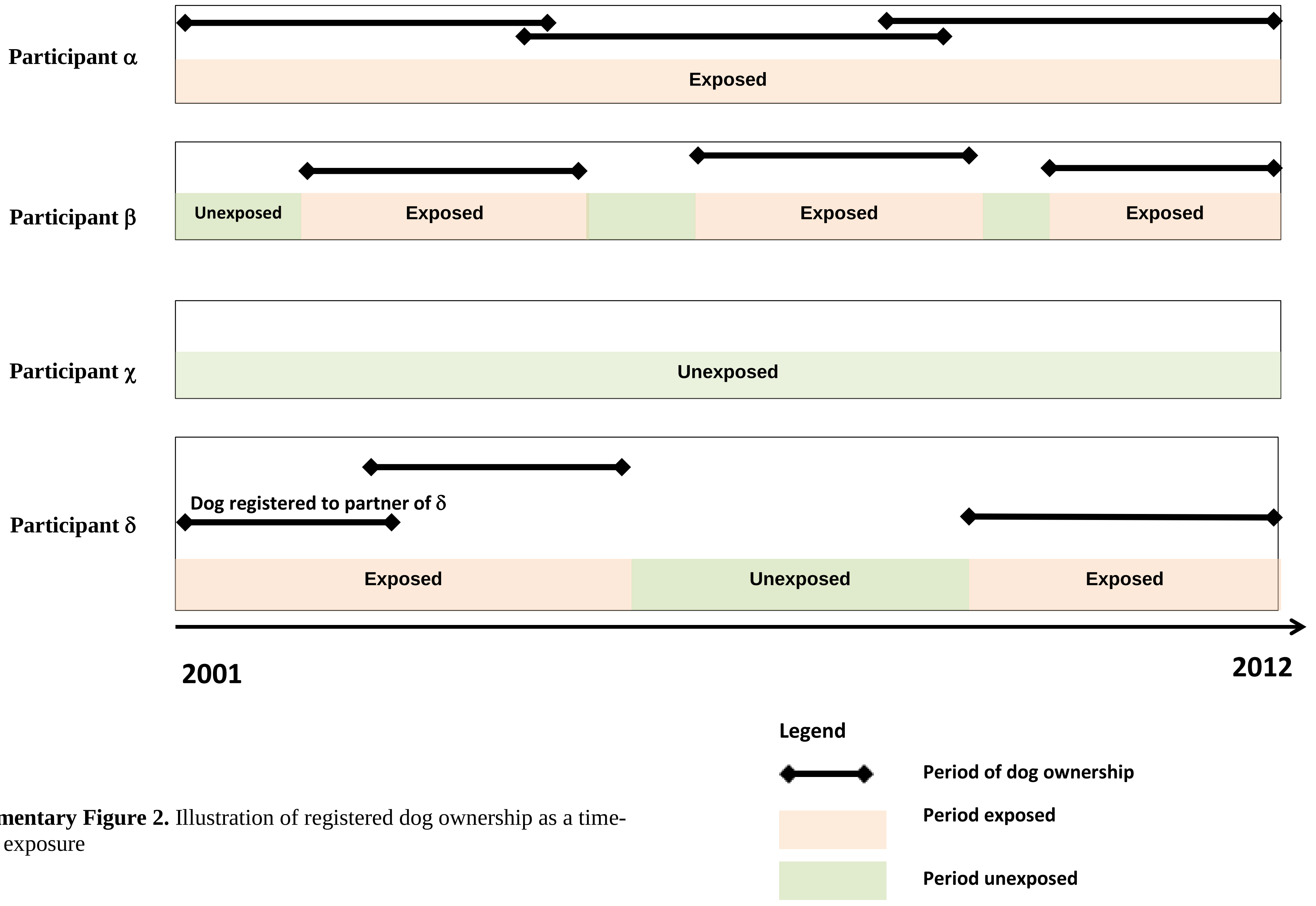

Supplementary Figure 2. Illustration of registered dog ownership as a time-varying exposure

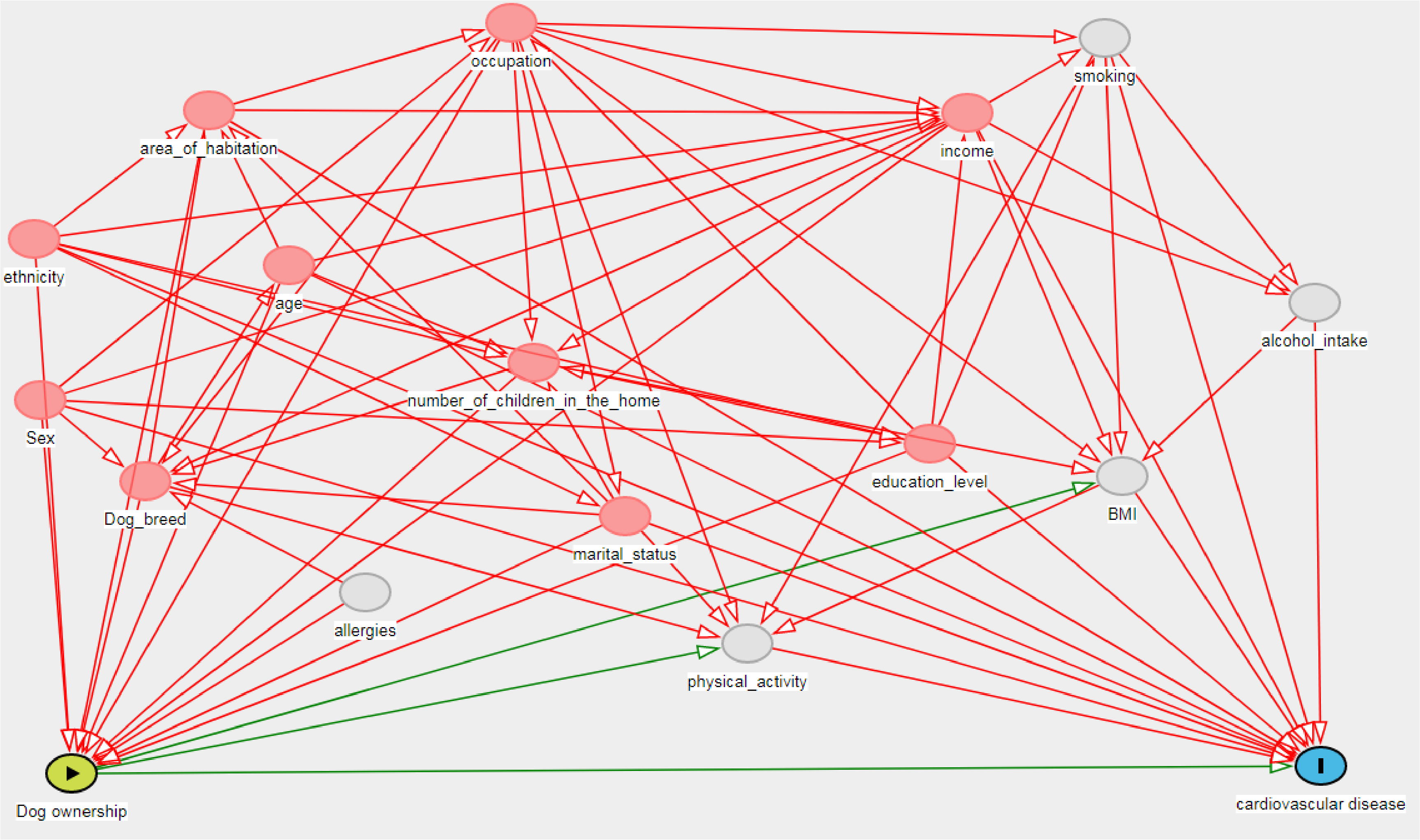

Supplementary Figure 3. Directed acyclic graph (DAG) for dog ownership and the risk of cardiovascular disease

References

1. Ludvigsson JF, Otterblad-Olausson P, Pettersson BU, Ekbom A. The Swedish personal identity number: possibilities and pitfalls in healthcare and medical research. European journal of epidemiology. 2009;24(11):659-67.
2. Lichtenstein P, De Faire U, Floderus B, Svartengren M, Svedberg P, Pedersen NL. The Swedish Twin Registry: a unique resource for clinical, epidemiological and genetic studies. J Intern Med. 2002;252(3):184-205.
3. Cederlof R, Lorich U. The Swedish Twin Registry. Prog Clin Biol Res. 1978;24 Pt B:189-95.
4. Lichtenstein P, Sullivan PF, Cnattingius S, Gatz M, Johansson S, Carlstrom E, et al. The Swedish Twin Registry in the third millennium: an update. Twin Res Hum Genet. 2006;9(6):875-82.
5. Charlson ME, Pompei P, Ales KL, MacKenzie CR. A new method of classifying prognostic comorbidity in longitudinal studies: development and validation. Journal of chronic diseases. 1987;40(5):373-83.
6. Quan H, Li B, Couris CM, Fushimi K, Graham P, Hider P, et al. Updating and validating the Charlson comorbidity index and score for risk adjustment in hospital discharge abstracts using data from 6 countries. American journal of epidemiology. 2011;173(6):676-82.
7. Rose D, Harrison E. The European socio-economic classification: a new social class schema for comparative European research. European Societies. 2007;9(3):459-90.
8. Ganzeboom HB, De Graaf PM, Treiman DJ. A standard international socio-economic index of occupational status. Social science research. 1992;21(1):1-56.
9. Kennelklubben S. Special Breed Specific Instructions (BSI) regarding exaggerations in pedigree dogs. Available: Accessed. 2014;1:3-7.
10. Bukodi E, Erikson R, Goldthorpe JH. The effects of social origins and cognitive ability on educational attainment. Acta Sociologica. 2014;57(4):293-310.
